# Hepatocyte Hypoxia Inducible Factor-1 Mediates the Development of Liver Fibrosis in a Mouse Model of Nonalcoholic Fatty Liver Disease

**DOI:** 10.1371/journal.pone.0168572

**Published:** 2016-12-28

**Authors:** Omar A. Mesarwi, Mi-Kyung Shin, Shannon Bevans-Fonti, Christina Schlesinger, Janet Shaw, Vsevolod Y. Polotsky

**Affiliations:** 1 Department of Medicine, University of California San Diego School of Medicine, San Diego, California, United States of America; 2 Department of Medicine, Johns Hopkins University School of Medicine, Baltimore, Maryland, United States of America; 3 The Joint Pathology Center, Bethesda, Maryland, United States of America; University of Navarra School of Medicine and Center for Applied Medical Research (CIMA), SPAIN

## Abstract

**Background:**

Obstructive sleep apnea (OSA) is associated with the progression of non-alcoholic fatty liver disease (NAFLD) to steatohepatitis and fibrosis. This progression correlates with the severity of OSA-associated hypoxia. In mice with diet induced obesity, hepatic steatosis leads to liver tissue hypoxia, which worsens with exposure to intermittent hypoxia. Emerging data has implicated hepatocyte cell signaling as an important factor in hepatic fibrogenesis. We hypothesized that hepatocyte specific knockout of the oxygen sensing α subunit of hypoxia inducible factor-1 (HIF-1), a master regulator of the global response to hypoxia, may be protective against the development of liver fibrosis.

**Methods:**

Wild-type mice and mice with hepatocyte-specific HIF-1α knockout (*Hif1a*^*-/-*^*hep*) were fed a high *trans*-fat diet for six months, as a model of NAFLD. Hepatic fibrosis was evaluated by Sirius red stain and hydroxyproline assay. Liver enzymes, fasting insulin, and hepatic triglyceride content were also assessed. Hepatocytes were isolated from *Hif1a*^*-/-*^*hep* mice and wild-type controls and were exposed to sustained hypoxia (1% O_2_) or normoxia (16% O_2_) for 24 hours. The culture media was used to reconstitute type I collagen and the resulting matrices were examined for collagen cross-linking.

**Results:**

Wild-type mice on a high *trans*-fat diet had 80% more hepatic collagen than *Hif1a*^*-/-*^*hep* mice (2.21 μg collagen/mg liver tissue, versus 1.23 μg collagen/mg liver tissue, p = 0.03), which was confirmed by Sirius red staining. Body weight, liver weight, mean hepatic triglyceride content, and fasting insulin were similar between groups. Culture media from wild-type mouse hepatocytes exposed to hypoxia allowed for avid collagen cross-linking, but very little cross-linking was seen when hepatocytes were exposed to normoxia, or when hepatocytes from *Hif1a*^*-/-*^*hep* mice were used in hypoxia or normoxia.

**Conclusions:**

Hepatocyte HIF-1 mediates an increase in liver fibrosis in a mouse model of NAFLD, perhaps due to liver tissue hypoxia in hepatic steatosis. HIF-1 is necessary for collagen cross-linking in an *in vitro* model of fibrosis.

## Introduction

Non-alcoholic fatty liver disease (NAFLD) is the most common disease of the liver in the West [1], and is the hepatic manifestation of the metabolic syndrome [2]. Some patients with NAFLD will see a progression of their disease to a phenotype of liver inflammation and fibrosis (non-alcoholic steatohepatitis, or NASH). In particular, hepatic fibrosis in NASH appears to lead to poorer outcomes, including need for liver transplantation and overall mortality [3]. Obstructive sleep apnea (OSA) is a common sleep disorder with an estimated prevalence of at least 4–5% in the general population [4]. The airway collapse that is the *sine qua non* of OSA has several downstream effects, including intermittent oxygen desaturations, arousals from sleep, and significant intrathoracic pressure swings [5]. In the last decade, a number of associative studies have noted that markers of liver injury and fibrosis in NAFLD are more pronounced in patients with severe OSA, independent of obesity, and this appears primarily related to the burden of nocturnal hypoxia [6–11]. This observation has now been made in several patient populations, both adult and pediatric. Moreover, chronic intermittent hypoxia (IH) mimicking the oxygen profile of patients with severe OSA in mice on a high fat diet also induces progression of hepatic steatosis to liver fibrosis and worsened hepatocellular injury [12, 13]. Despite the increasingly robust evidence of an associative link between NAFLD progression and OSA severity, there has been comparatively little investigation to suggest a mechanism which may tie together any aspect of these two illnesses.

In NAFLD, hepatic stellate cell activation has been proposed as a crucial step in the progression toward hepatic fibrosis, and hepatocyte injury and apoptosis have been noted in this progression as well [14, 15]. However, little data is available about the importance of tissue hypoxia in the hepatocyte in NAFLD. Emerging evidence has demonstrated that liver hypoxia may be a key mediator of several diseases of the liver [16], and our approach was to consider the possibility that hepatocyte hypoxia may be a key mechanistic step needed for OSA to impact the progression of NAFLD. A number of recent publications have demonstrated that tissue hypoxia, among other stimuli, increases expression of the fibrogenic enzyme lysyl oxidase (LOX) [17, 18], regulated by hypoxia inducible factor-1 (HIF-1) [17, 19, 20]. LOX is a secreted amine oxidase which catalyzes the formation of covalent cross-links between collagen fibers in the extracellular matrix [21]. There is emerging evidence that lysyl oxidase and related proteins may be implicated in the development of hepatic fibrosis [22, 23]. We have previously shown that hypoxia increases LOX gene expression in cultured mouse hepatocytes [24], although we have not previously investigated the role of HIF-1 in this process. Additionally, we have shown that in bariatric patients, serum LOX is elevated in patients with NAFLD-associated hepatic fibrosis, relative to those without fibrosis [24]. In mice, high fat diet-induced fatty liver leads to liver tissue hypoxia [25, 26], and is associated with increased hepatic expression of HIF-1α, the oxygen sensitive subunit of HIF-1 [27, 28]. This finding is not present in mice on a regular chow diet [28].

Based on these observations, we sought to apply our data to a mouse model, utilizing a high *trans*-fat diet (HTFD) to induce NASH, as previously described [29, 30]. We hypothesized that liver hypoxia in this NAFLD model would lead to HIF-1 activation, and enhanced liver fibrosis via LOX upregulation. Furthermore, we hypothesized that knockout of HIF-1α in the hepatocyte—by use of a Cre-recombinase system tied to an albumin promoter—would reduce liver fibrosis in our model. To test our hypothesis, we performed a set of experiments. First, we isolated hepatocytes from mice with knockout of HIF-1α and wild-type control, and observed collagen cross-linking in response to hypoxia. Second, we fed mice from both groups a HTFD for six months, so as to observe differences in metabolic outcomes and liver fibrosis.

## Methods

### Mice

Mice with HIF-1α knockout specific to hepatocytes (*Hif1a*^*-/-*^*hep* mice) were generated as follows: B6.129-*Hif1a*^*tm3Rsjo*^/J mice (*Hif1a*^*F/F*^) from The Jackson Laboratory (Bar Harbor, ME, stock #007561) were mated to B6.Cg-Tg(*Alb-cre*)21Mgn/J mice (*Alb-cre* mice) from the Jackson Laboratory (stock #003574). In *Alb-cre* mice, the Cre-recombinase gene is driven by the albumin promoter resulting in hepatocyte specific expression of Cre-recombinase and hence gene knockout in hepatocytes [26, 31]. *Hif 1a*^*F/F*^*Alb-Cre*^*+/+*^ and *Hif 1a*^*F/F*^*Alb-Cre*^*-/-*^ mice from the same colony were used as *Hif1a*^*-/-*^*hep* and wild-type controls, respectively. For *in vitro* experiments, eight week old *Hif1a*^*-/-*^*hep* and wild-type mice were fed a chow diet and were exposed to light from 9 am until 9 pm daily. For *in vivo* experiments, eight week old *Hif1a*^*-/-*^*hep* (n = 15) and wild-type mice (n = 13) were fed a HTFD for six months. Animals were housed 3–5 per cage and food intake and weight were recorded twice weekly. Food intake was calculated as the average per mouse based on the difference between original food weight and food remaining, at each weighing. At sacrifice, serum and liver and epididymal fat were collected. Animal studies were approved by the Institutional Animal Care and Use Committee of the Johns Hopkins University School of Medicine. All mouse surgery and euthanasia was performed with isoflurane anesthesia (1–2%), and all efforts were made to minimize suffering.

### Hepatocyte isolation and hypoxia exposure

For *in vitro* experiments, hepatocytes were isolated from adult mice (n = 3 per group) using a two-step perfusion process [32], then plated onto dishes with Dulbecco’s Modified Eagle Medium containing high glucose, L-glutamine and phenol red (Life Technologies, Grand Island, NY), with 10% fetal bovine serum. The media was replaced with serum-free media the following day. The cells were placed for 24 hours into airtight chambers containing 5% CO_2_ and either 1% or 16% O_2_. After exposure, cell media was collected and cells were recovered and stained with 0.4% Trypan Blue solution (Life Technologies, Rockville, MD) to quantify hepatocyte survival.

### RT-PCR and western blot

Total RNA was isolated from hepatocytes exposed to hypoxia and from liver tissue. RNA was extracted from livers using Trizol reagent (Life Technologies, Rockville, MD). cDNA was synthesized using an Advantage RT for PCR kit (Clontech, Palo Alto, CA). Real-time reverse transcriptase PCR (RT-PCR) was performed with primers from Invitrogen (Carlsbad, CA), and Taqman probes from Applied Biosystems (Foster City, CA). Target mRNA level was normalized to 18s rRNA, using the formula: Target/18s = 2^Ct(18s)–Ct(target)^. Western blotting was performed with the primary antibody to HIF-1α (rabbit polyclonal, 1:1000, Novus Biologicals, Littleton, CO) and to HIF-2α (mouse monoclonal, 1:2000, Abcam, Cambridge, MA).

### Collagen assay

A collagen cross-linking assay similar to one previously described [19] was constructed in order to determine whether hypoxia may create an environment more conducive to collagen cross-linking. A stock solution of type I rat tail collagen at 3 mg/mL (Life Technologies, Grand Island, NY) was diluted to a final concentration of 1.2 mg/mL with 300 μL of concentrated culture media from hepatocytes isolated from *Hif1a*^*-/-*^*hep* and wild-type mice, and double distilled water, then plated onto coverslip-bottom plastic dishes (MatTek Corp., Ashland, MA), and incubated at 37°C for 16 hours. The resulting collagen matrix was examined using confocal reflection microscopy with a Leica TCS SP5 microscope and the accompanying Leica Application Suite software (Wetzlar, Germany).

### Intraperitoneal glucose tolerance test (IPGTT) and insulin tolerance test (ITT)

IPGTT and ITT were performed two weeks and one week prior to mouse sacrifice, respectively. IPGTT was performed after a 5-hour fast by injecting 1 g/kg glucose intraperitoneally. Glucose levels were measured by tail-snip technique using a handheld glucometer (ACCU-CHECK Aviva Plus, Roche, Indianapolis, IN) at baseline, and at 10, 20, 30, 60, 90, and 120 minutes after glucose injection. ITT was performed after a 5-hour fast by injecting 0.5 IU/kg insulin (Humulin R, Eli Lilly, Indianapolis, IN) intraperitoneally. Glucose levels were measured similarly to the IPGTT, at 10, 20, 30, 40, 50, 60, 90, and 120 minutes after insulin injection. IPGTT and ITT were analyzed by subtracting fasting glucose level and calculating area under the curve for each mouse.

### Immunohistochemistry

Fresh liver tissue samples were collected in 10% buffered formalin, and then dehydrated with 70% ethyl alcohol after 24 hours, and embedded in paraffin. Liver tissue was sectioned into 5 μm slices for immunohistochemistry. The Hypoxyprobe-1 kit (Hypoxyprobe, Inc., Burlington, MA) was used for piminidazole staining, according to the manufacturer’s protocol. Briefly, for two mice per group, to confirm liver tissue hypoxia, pimonidazole was injected intraperitoneally at 60 mg/kg 60 minutes before tissue collection. Following injection, pimonidazole distributes to all tissues except brain, and forms adducts with thiol containing proteins only in cells with oxygen tension less than 10 mm Hg at 37°C. Immunostaining was performed with the primary Hypoxyprobe-1 antibody per package insert, and a secondary antibody of biotin-conjugated F(ab’)_2_, followed by addition of streptavidin peroxidase and 3,3’-diaminobenzidine (Vector Laboratories, Burlingame, CA) as a peroxidase substrate. These mice were not included into analyses for liver or metabolic outcomes and were used only to qualitatively assess liver hypoxia. Immunohistochemistry for LOX was performed with primary antibody to LOX (rabbit polyclonal, 1:200, Novus Biologicals, Littleton, CO).

### Serum insulin and liver enzymes, hepatic triglyceride, and hepatic LOX quantification

Serum insulin was measured with a Mouse Ultrasensitive ELISA Kit (Alpco, Salem, NH). Liver enzymes were analyzed at the Johns Hopkins Bayview Medical Center. Lipids were extracted from the liver by using a standard chloroform-methanol extraction technique (the Folch method). Liver triglycerides were measured with a kit from Wako Diagnostics, Inc. (Richmond, VA). Hepatic LOX levels were assessed by LOX activity assay kit (Abcam, Cambridge, MA).

### Sirius red staining and hydroxyproline quantification

Paraffin-embedded tissue samples were used to make 5 μm sections for staining for Sirius red staining using a commercially available kit (Abcam, Cambridge, MA). 12 random images from each slide were obtained and these images were analyzed by measuring the percentage of each image for which Sirius red staining was determined to be above an arbitrary threshold in a blinded fashion, using Metamorph Advanced Acquisition software (Molecular Devices, LLC, Sunnyvale, CA). The threshold area for each mouse sample was determined as the average of the value from the 12 images per slide. Hydroxyproline was quantified by use of commercial assay (QuickZyme Biosciences, Leiden, Netherlands). Collagen was determined from this assay by assuming a hydroxyproline content of 13.5% [33], and samples were normalized for liver weight.

### Statistical analysis

For single point measurement, statistical comparisons between groups of mice were performed using an unpaired *t*-test with Bonferroni correction for multiple comparisons. For multiple measurements, a repeated measure analysis of variance was performed. For all statistical comparisons, a p-value <0.05 was the threshold used for statistical significance. Data are reported as mean ± SEM unless otherwise noted. Stata 12 software (StataCorp, College Station, TX) was used for all analyses.

## Results

### Hypoxia *in vitro* leads to LOX overexpression via HIF-1

**Fig 1:**
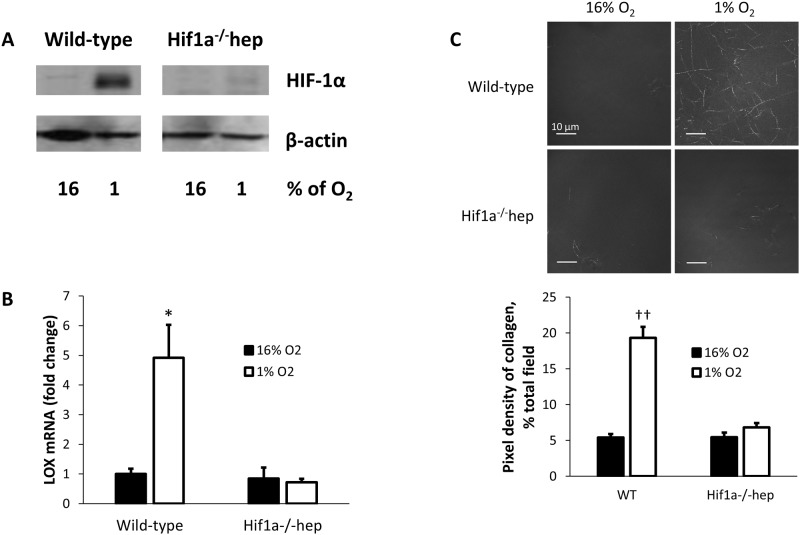
Hypoxia accentuates LOX expression via HIF-1, leading to enhanced collagen cross-linking. (A) Hypoxic exposure increases expression of HIF-1α in the nuclear extract of wild-type mouse hepatocytes. This change is abolished in hepatocytes from *Hif1a*^*-/-*^*hep* mice. (B) LOX mRNA expression increases in wild-type hepatocytes exposed to hypoxia, but this increase in LOX is not observed in *Hif1a*^*-/-*^*hep* hepatocytes. (C) When culture media from wild-type hepatocytes is added to collagen, hypoxia causes increased collagen cross-linking and precipitation; this difference is not seen in *Hif1a*^*-/-*^*hep* hepatocytes. *, p<0.05; ††, p<0.001.

Primary hepatocytes were exposed to normoxia or hypoxia (16% or 1% O_2_ and 5% CO_2_), serving as a model for the hypoxia induced by hepatic steatosis [25, 26], and other diseases of hypoxia, such as OSA. Wild-type mouse hepatocytes increased HIF-1α protein levels in hypoxia but not in normoxia. No significant HIF-1α was found in hepatocytes from *Hif1a*^*-/-*^*hep* mice in either condition (Fig 1A). Similarly, LOX was overexpressed in wild-type hepatocytes exposed to hypoxia, but not in normoxia or in HIF-1α knockout, demonstrating that hepatocyte LOX is increased in hypoxia *via* HIF-1 (Fig 1B). We have previously shown that overexpression of LOX in hypoxia results in enhanced LOX protein secretion to the media of cultured hepatocytes, and thus, enhanced collagen cross-linking [22]. This paradigm was reapplied to hepatocytes from each mouse strain. We found that culture media from wild-type hepatocytes robustly cross-linked collagen only in hypoxia, but this effect was abolished entirely when culture media from *Hif1a*^*-/-*^*hep* hepatocytes was used (Fig 1C). Thus, hypoxia results in more avid collagen cross-linking, *via* hepatocyte HIF-1.

### Mouse characteristics

**Fig 2:**
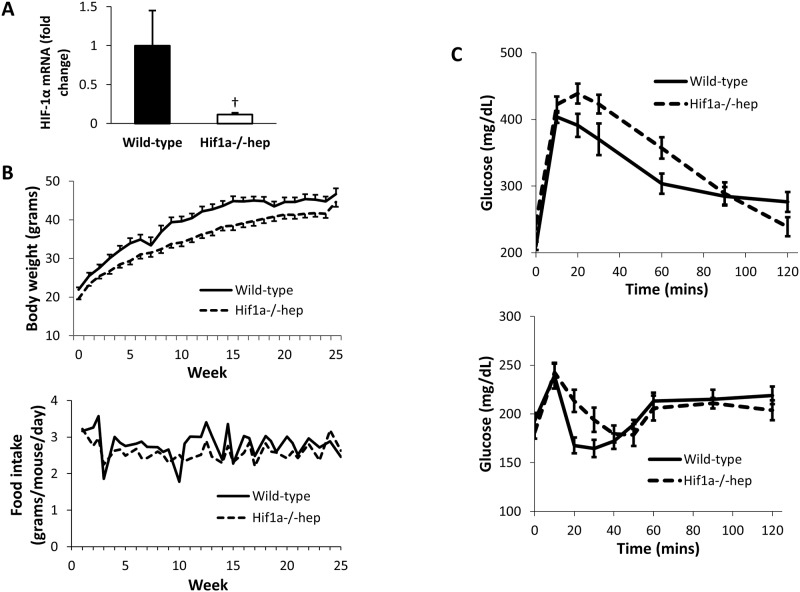
Metabolic characteristics of wild-type and *Hif1a*^*-/-*^*hep* mice. (A) Hepatocyte knockout of HIF-1α was confirmed by quantifying HIF-1α mRNA expression from the nuclear extract of isolated hepatocytes. (B) Body weight (top) and food intake (bottom) over experiment duration. (C) IPGTT (top) and ITT (bottom) at time of sacrifice. †, p<0.005.

Mice of each genotype were fed a HTFD for six months, without weight matching. Knockout of HIF-1α in the hepatocyte from the knockout group was confirmed by RT-PCR of liver tissue from *Hif1a*^*-/-*^*hep* and wild-type mice, which showed a decrease in HIF-1α expression of 88% (p = 0.002, Fig 2A). Mice in each group were metabolically similar. They gained weight at a similar rate, and food intake was not different between groups (Fig 2B). At sacrifice at six months, body weight was similar between groups (46.7 ± 1.5 g for wild-type mice, and 44.6 ± 1.2 g for *Hif1a*^*-/-*^*hep* mice, p = 0.289), as was liver weight (4.7 ± 0.4 g for wild-type mice, and 4.7 ± 0.3 g for *Hif1a*^*-/-*^*hep* mice, p = 0.972). Fasting blood glucose and insulin levels were similar between groups (glucose: 201.6 ± 6.2 mg/dL for wild-type mice, and 212.5 ± 6.4 mg/dL for *Hif1a*^*-/-*^*hep* mice, p = 0.239; insulin: 0.662 ± 0.073 ng/mL for wild-type mice, and 0.636 ± 0.060 ng/mL for *Hif1a*^*-/-*^*hep* mice, p = 0.781). Glucose tolerance was similar (Fig 2C; AUC for wild-type group 12,934 ± 1335 mg*min/dL, and for *Hif1a*^*-/-*^*hep* group 12,007 ± 1469 mg*min/dL, p = 0.647), as was insulin sensitivity (AUC for wild-type group 12,712 ± 482% baseline*min, and for *Hif1a*^*-/-*^*hep* group 13,714 ± 751 mg*min/dL, p = 0.280).

### High fat diet induces hepatic hypoxia and HIF-1 activation

**Fig 3:**
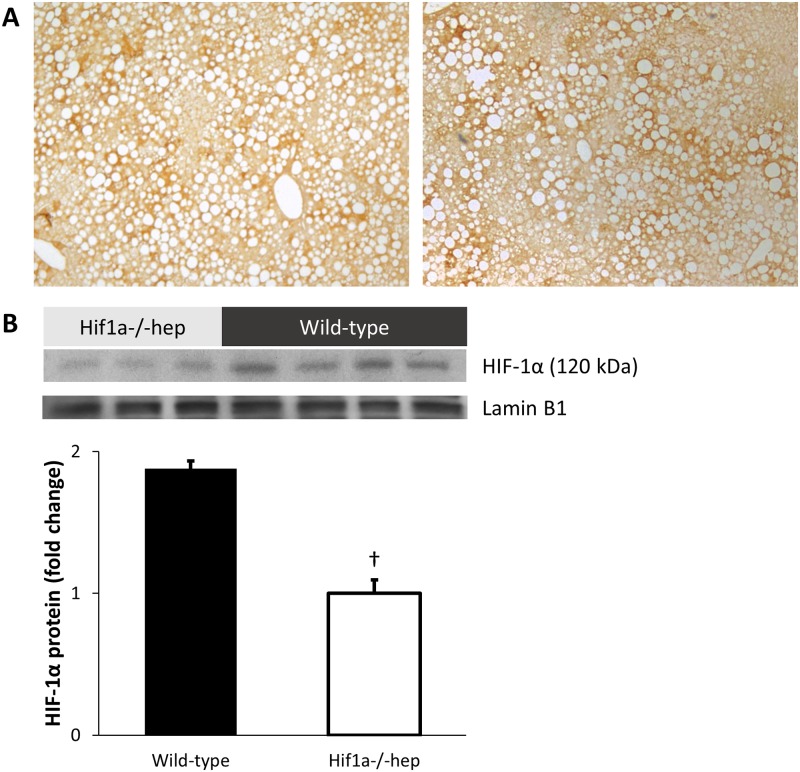
Liver hypoxic profile. (A) Wild-type (left) and *Hif1a*^*-/-*^*hep* (right) liver tissue was similarly hypoxic after six months on an HTFD, as qualitatively assessed by Hypoxyprobe stain. (B) As expected, HIF-1α protein levels were reduced in *Hif1a*^*-/-*^*hep* mice. Some HIF-1 activation in *Hif1a*^*-/-*^*hep* mouse liver tissue likely remains due to incomplete gene knockout, and preserved HIF-1α in non-hepatocyte cell types. †, p<0.005.

Positive Hypoxyprobe-1 staining, indicative of severe tissue hypoxia, was found in both groups of mice (Fig 3A). HIF-1α was activated in wild-type mice (Fig 3B), which was less evident in the hepatocyte knockout group: HIF-1α protein was increased 1.9-fold in wild-type mice, p = 0.003. HIF-2α was similar between groups (p = 0.218, S1 Fig).

### Liver fibrosis is reduced in *Hif1a*^*-/-*^*hep* mice

**Fig 4:**
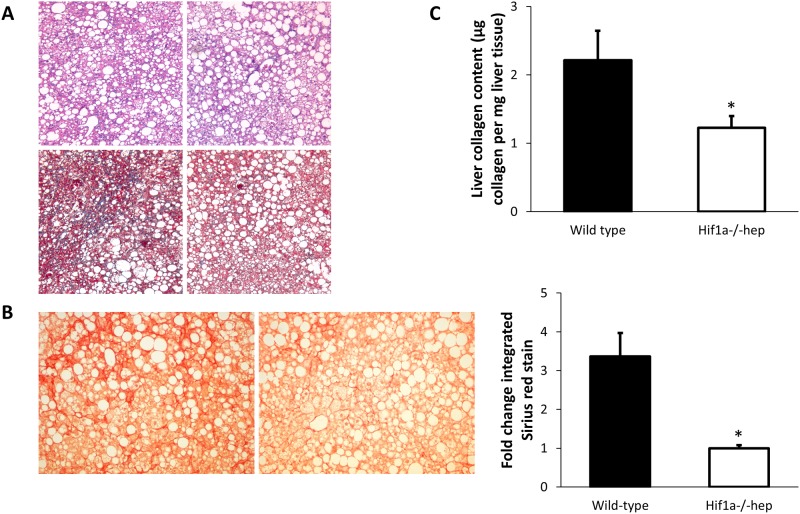
Liver histology and collagen quantification. (A) Representative liver H&E (top) and Masson’s trichrome stains (bottom) from wild-type (left) and *Hif1a*^*-/-*^*hep* mice. More fibrosis can be observed in the wild-type mice in the Masson’s trichrome stain. (B) Sirius red stain of collagen in wild-type (left) and *Hif1a*^*-/-*^*hep* mice (right). (C) Collagen content of all samples by use of hydroxyproline assay. *, p<0.05.

After six months on the HTFD, mice from both groups exhibited marked macrovesicular steatosis (Fig 4A, Table 1). Liver triglyceride content was similar in both groups (46.7 ± 4.4 mg/g liver tissue in wild-type mice, and 38.8 ± 3.7 mg/g liver tissue in *Hif1a*^*-/-*^*hep* mice, p = 0.201). Serum liver enzymes were also elevated in both groups of mice, in a manner similar to previous reports [29, 30]. Aspartate aminotransferase was not different between groups (412 ± 49 U/L in wild-type mice, and 522 ± 54 U/L in *Hif1a*^*-/-*^*hep* mice, p = 0.153), but alanine aminotransferase (357 ± 23 U/L in wild-type mice, and 639 ± 92 U/L in *Hif1a*^*-/-*^*hep* mice, p = 0.014) and alkaline phosphatase (216 ± 6 U/L in wild-type mice, and 171 ± 10 U/L in *Hif1a*^*-/-*^*hep* mice, p = 0.002) were different between groups. Total bilirubin and gamma-glutamyl transpeptidase levels were normal in all mice, and were not different between groups.

**Table 1:** Mouse Histologic Characteristics.

Mouse ID	Steatosis %	Activity Grade	Ballooning
WT 1	90–95% (small droplet > macro)	1	no
WT 2	85–90% (small droplet > macro)	1	no
WT 3	80–85% (small droplet > macro)	2	yes
WT 4	70–75% (small droplet >> macro)	1	no
WT 5	80–85% (small droplet = macro)	2	yes
WT 6	80–85% (small droplet > macro)	1	no
WT 7	80–85% (small droplet = macro)	3	yes
			
*Hif1a*^*-/-*^*hep* 1	90–95% (small droplet >> macro)	2	yes
*Hif1a*^*-/-*^*hep* 2	90–95% (small droplet = macro)	1	yes
*Hif1a*^*-/-*^*hep* 3	90–95% (small droplet > macro)	2	yes (Nod Reg)
*Hif1a*^*-/-*^*hep* 4	80–85% (small droplet = macro)	2	yes
*Hif1a*^*-/-*^*hep* 5	90–95% (small droplet = macro)	2	yes
*Hif1a*^*-/-*^*hep* 6	80–85% (small droplet > macro)	1	no
*Hif1a*^*-/-*^*hep* 7	80–85% (small droplet = macro)	3	yes
*Hif1a*^*-/-*^*hep* 8	90–95% (small droplet >> macro)	2	yes
*Hif1a*^*-/-*^*hep* 9	90–95% (small droplet > macro)	1	no
*Hif1a*^*-/-*^*hep* 10	90–95% (small droplet > macro)	1	no

**Fig 5:**
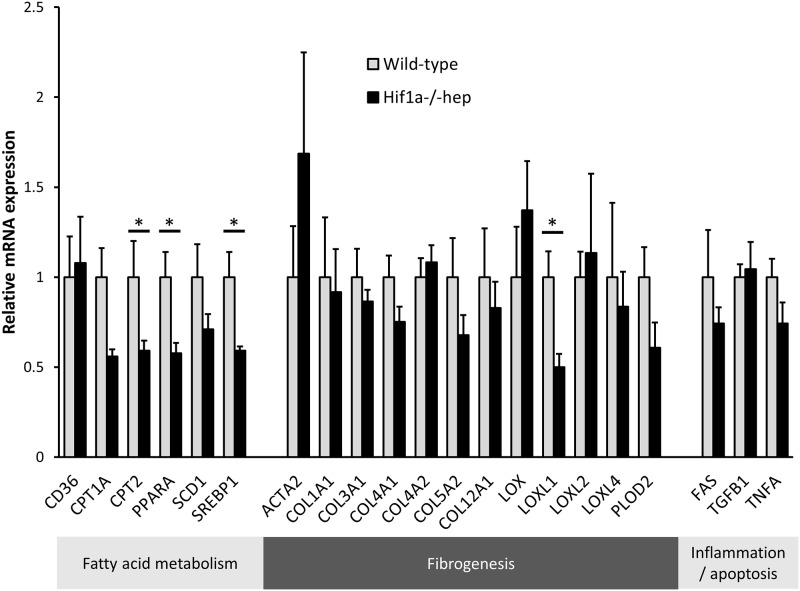
Expression of genes involved in fibrosis, hepatic lipid metabolism, and inflammation/apoptosis.

Sirius red staining of mouse livers demonstrated that *Hif1a*^*-/-*^*hep* mice had markedly reduced liver fibrosis compared to wild-type controls (Fig 4B). Quantitative analysis of Sirius red images showed that *Hif1a*^*-/-*^*hep* mice had a 74% reduction in collagen staining (1.01 ± 0.21% of image with positive Sirius red stain in wild-type mice, and 0.26 ± 0.03% in *Hif1a*^*-/-*^*hep* mice, p = 0.011). Hydroxyproline content, a surrogate of hepatic collagen content, was reduced in *Hif1a*^*-/-*^*hep* mice (2.21 ± 0.43 μg/mg collagen in wild-type mice, and 1.23 ± 0.17 μg/mg in *Hif1a*^*-/-*^*hep* mice, p = 0.030, Fig 4C). Hepatic LOX activity was slightly increased in wild-type mice relative to *Hif1a*^*-/-*^*hep* mice (1.09-fold increased LOX activity, p = 0.046). However, there was no difference in mRNA expression of LOX between groups. We also queried other members of the LOX-like family to see if there was a difference in expression between genotypes. Hepatic LOXL2 and LOXL4 were unchanged between wild-type and *Hif1a*^*-/-*^*hep* mice, although there was a 42% reduction in LOXL1 among *Hif1a*^*-/-*^*hep* mice (p = 0.023, Fig 5). Despite this finding, there was no significant difference in hepatic LOXL1 protein content between groups. Collagen subtypes were unchanged between groups, as were other genes involved in fibrogenesis. Although hepatic triglyceride content was similar between genotypes, there were differences in several genes involved in hepatic fatty acid metabolism (CPT2, PPARA, and SREBP1).

## Discussion

In this study we have examined the role that hepatocyte HIF-1 may play in the development of liver fibrosis in a mouse model of NAFLD. We have confirmed previous findings that a HTFD results in the development of NASH in wild-type mice, with significant hepatic fibrosis. The main novel finding of our study is that hepatocyte knockout of HIF-1α results in a marked reduction in hepatic fibrosis, suggesting that hepatocyte HIF-1 is implicated in the development of liver fibrosis in the mouse model of NAFLD.

Several studies have shown that liver fibrogenesis may be related to tissue hypoxia. Liver injury may lead to tissue hypoxia in NAFLD [34], perhaps due to changes in perfusion of the liver or mitochondrial modifications [24]. Consistent with our present findings, others have reported activation of HIF-1 in mouse models of NAFLD [24, 25]. HIF-1 is a heterodimer which consists of a constitutively expressed β subunit and an O_2_-regulated α subunit. HIF-1 activation generally occurs due to the inhibition of O_2_-dependent prolyl hydroxylation, thereby preventing ubiquitination and proteasomal degradation [35, 36]. Several groups have explored the link between HIF-1 and liver fibrosis in animal models. Copple *et al*. have shown that hypoxia activates HIF-1α in isolated hepatocytes, and that this leads to increases in various profibrotic mediators [37]. They also showed that, in isolated hepatic stellate cells, hypoxia activated both HIF-1α and HIF-2α, leading to increases in profibrotic gene expression [38], and that deletion of HIF-1α in Mx interferon-expressing cells resulted in reduced liver fibrosis in a bile duct ligation model of cholestatic liver injury, via dysregulation of similar profibrotic mediators [39]. Qu *et al*. have found that HIF-2α mediates an increase in lipid accumulation in mice on a chow diet, and that it results in hepatic fibrosis in mice on an alcohol diet [40]. HIF-2 signaling was shown to have a significant impact on hepatic lipid metabolism and glucose handling in mice on a high fat diet [41]. These lines of evidence collectively support the idea of tissue hypoxia as a “second hit” that may lead to fibrosis in a susceptible liver. Our study provides the first evidence that hypoxia contributes to the development of liver fibrosis in NAFLD, acting *via* HIF-1 in hepatocytes. Hepatocyte HIF-2 signaling may still have important downstream effects with respect to hepatic fibrosis or other pathophysiologic features in NAFLD, but this has yet to be determined.

Mechanisms by which hepatocyte HIF-1 may mediate liver fibrosis in NAFLD remain unclear. Our query into LOX was based on data that showing that LOX, which has hypoxia response elements in its promoter region, is a target of HIF-1 in various circumstances of tissue hypoxia [42–44]. We have recently shown that hypoxic hepatocytes overexpress and secrete LOX, causing increased collagen cross-linking [22], and our current data in vitro add to this by demonstrating the HIF-1-dependency of this phenomenon. Although our cell culture data implicated LOX as a mediator of enhanced collagen cross-linking acting *via* hypoxia and HIF-1, our mouse experiment did not show similar findings. HIF-1 also did not seem to act *via* LOXL2 or LOXL4, nor through enhanced collagen gene expression or hepatic stellate cell activation (based on α-smooth muscle actin expression) [45]. A difference in LOXL1 gene expression between groups did not translate to a difference in protein concentration, and therefore it is less likely that LOXL1 is a significant mediator of the hepatocyte HIF-1 effect on liver fibrosis.

There are several reasons for an apparent discrepancy between in vitro and in vivo findings. First, It is conceivable that HIF-1-dependent LOX changes occur earlier in the time course. Second, HIF-1α knockout was specific for hepatocytes, whereas other liver cell types, for example stellate cells, could still express LOX; it would suggest that the difference in fibrosis between HIF-1α hepatocyte genotypes was LOX-independent. Third, HIF-1 may also modulate hepatic inflammation, which could contribute to fibrogenesis [46, 47].

Interestingly, although hepatic triglyceride content was similar between genotypes, several genes of fatty acid metabolism were different between groups. Since two of these enzymes (CPT2 and PPARA) regulate FA oxidation and utilization, and the other (SREBP1) regulates lipid synthesis, we hypothesize that: a) that these disparate factors roughly balance out; b) that the effects are not large enough in any single direction to cause a noticeable difference in hepatic triglycerides; and/or c) that there may be a “ceiling effect” of hepatic triglyceride content such that minor changes in FA synthesis or utilization/oxidation genes are insufficient to significantly impact hepatic triglyceride content at these levels, at least at this time point when profound hepatic steatosis has already been established.

There are several limitations to our study. First, our model is one of NASH and not just of NAFLD. We chose this particular diet so as to mimic a severe phenotype of hepatic injury in NAFLD, and to induce hepatic fibrosis in a relatively short time course. This model has advantages over other models of liver fibrosis in that it is diet-based, but the degree of liver injury may not be a realistic representation of typical human NAFLD. To this point, liver enzymes in our mice were highly elevated, and hepatocellular injury was more prominent among knockout mice (as evident from the ALT increase), even as biliary injury was less in the wild-type mice (as evident from the alkaline phosphatase increase). We do not have a clear explanation for why this might be since there is little data on the importance of HIF-1 signaling specifically in the biliary system. Second, our model allows us to query the significance of HIF-1 in hepatocytes, but this approach does not take into account the significance of HIF-1 signaling in other cell types, such as endothelial cells and hepatic stellate cells, and the importance of the other major hypoxia inducible factor, HIF-2. Finally, the mechanism facilitating the development of liver fibrosis downstream of hepatocyte HIF-1 is yet to be elucidated.

In conclusion, we have found that hepatocyte HIF-1 may play an important role in the development of fibrosis in a model of NAFLD-induced liver damage, and we suggest that this may mechanistically implicate hypoxia as a key driver toward liver injury in NAFLD. This has important ramifications in patients with NAFLD who also have other co-morbidities predisposing them to severe hypoxia, such as in OSA. We speculate that liver fibrosis in patients with OSA and NAFLD may be linked through hepatocyte hypoxia, that this may account for a shift in NAFLD phenotype among OSA patients toward more severe disease, and that further experiments are needed to determine the role of aggressive diagnosis and treatment of OSA among NAFLD patients.

## Supporting Information

S1 Fig HIF-2 protein levels. HIF-2 protein levels were not different between *Hif1a*^*-/-*^*hep* and wild-type mice, suggesting that phenotypic differences seen between groups are not likely due to this other major hepatic HIF isoform.
(TIF)

S1 File Supporting data for all figures. (XLSX)
