# Supplementary figures and images for: Hepatocyte Hypoxia Inducible Factor-1 Mediates the Development of Liver Fibrosis in a Mouse Model of Nonalcoholic Fatty Liver Disease
Source: PLoS One. 2016 Dec 28;11(12):e0168572. doi: 10.1371/journal.pone.0168572 (PMC5193414; doi:10.1371/journal.pone.0168572)

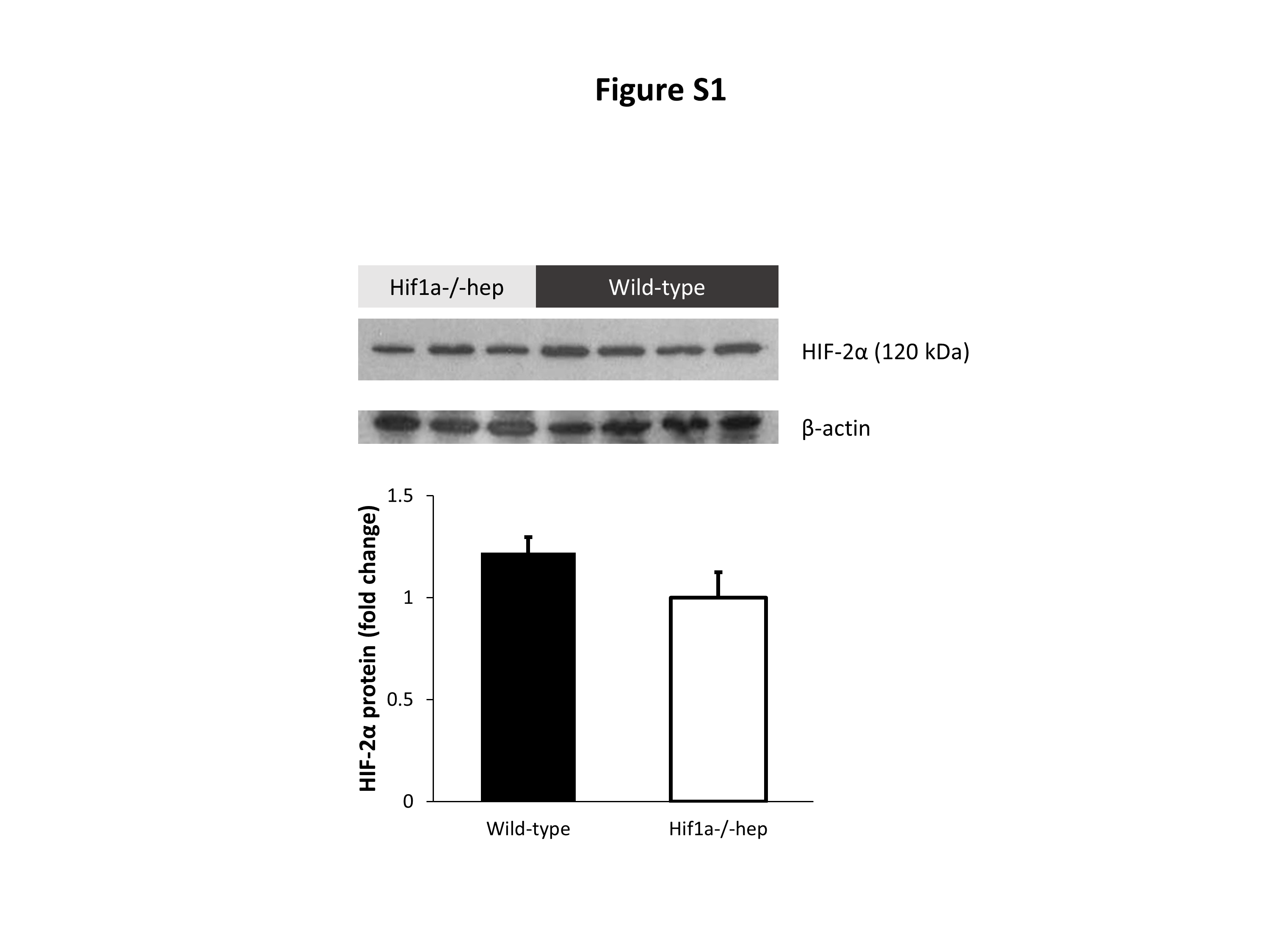

Supplement: S1 Fig — HIF-2 protein levels were not different between Hif1a-/-hep and wild-type mice, suggesting that phenotypic differences seen between groups are not likely due to this other major hepatic HIF isoform. (TIF) [file pone.0168572.s001.TIF]
